# Incidence and risk factors of unplanned retreatment following dental general anesthesia in children with severe early childhood caries

**DOI:** 10.3389/fped.2023.1163368

**Published:** 2023-07-27

**Authors:** Jin-yi Li, Shu-yang He, Pan-xi Wang, Shan-shan Dai, Shu-qi Zhang, Zheng-yang Li, Qing-yu Guo, Fei Liu

**Affiliations:** ^1^ Key Laboratory of Shaanxi Province for Craniofacial Precision Medical Research, College of Stomatology, Xi’an Jiaotong University, Xi’an, China; ^2^ Department of Pediatric Dentistry, Afﬁliated Stomatology Hospital of Xi’an Jiaotong University, Xi’an, China; ^3^ Faculty of Dentistry, The University of Hong Kong, Hong Kong SAR, China

**Keywords:** dental general anesthesia, unplanned retreatment, survival analysis, severe early childhood caries, primary tooth

## Abstract

**Objective:**

This study aimed to retrospectively describe the unplanned retreatment of dental general anesthesia (DGA) in children with severe early childhood caries (S-ECC) and explore potential factors that may influence the outcome of DGA treatment.

**Methods:**

Medical records of children with S-ECC who received DGA treatment were screened, and necessary data were extracted. The Kaplan–Meier method and Cox proportional hazards model were used to estimate the DGA survival rate and explore the potential factors affecting the success rate of DGA treatment.

**Results:**

Medical records of 852 children were included; 509 (59.7%) children with 1,212 (10.7%) teeth underwent unplanned retreatment. Restoration failure (30.12%) and new caries (29.46%) accounted for the most significant proportion of all failures. The median survival times were 510 and 1,911 days at the child and tooth levels, respectively. Unplanned retreatment risk was associated with the age of S-ECC children, frequency of follow-up, and fluoride application (hazard ratio = 0.97, 0.78, 0.69, *P *< 0.001).

**Conclusion:**

The treatment outcome of DGA administered to children with S-ECC was satisfactory at the tooth level from the perspective of the incidence of unplanned retreatment. Restoration failure was the main reason for the high unplanned retreatment rate. Strategies for a better outcome of DGA include improving the professional knowledge and skills of pediatric dentists and enhancing compliance of parents/patients. Health education and regular topical fluoride application may improve the success rate of DGA treatment.

## 1. Introduction

Dental caries is one of the most prevalent chronic infectious childhood diseases (1). Dental caries and pulp diseases are associated with undernutrition, systemic diseases, and, in some cases, life-threatening problems (2). Oral problems in childhood significantly affect not only the oral health of children but also their general health and quality of life (3). Although the prevention and management of primary tooth caries have been conducted for several years, it remains a significant public health concern, and the burden varies unequally across countries (4). Furthermore, because of the socioeconomic factors and physiological features of primary teeth, dental caries in children can easily progress to severe early childhood caries (S-ECC). The S-ECC consists of three conditions: (1) any sign of smooth-surface tooth caries in children less than 3 years old; (2) one or more cavitated, missing, or filled smooth surfaces in primary maxillary anterior teeth from ages 3–5 years; and (3) a decayed, missing, or filled score of ≥4 (age 3), ≥5 (age 4), or ≥6 (age 5) surfaces (5). Treatment and management of S-ECC is expensive and usually requires extensive restorative treatment or tooth extraction. However, children with S-ECC who usually experience dental fear and cannot cooperate with dentists cannot endure complex procedures. This poses a significant challenge for pediatric dentists when the behavioral management of pediatric patients is ineffective.

The American Academy of Pediatric Dentistry (AAPD) recommends dental general anesthesia (DGA) as an alternative pharmacological intervention for S-ECC patients when other behavioral management methods are ineffective (6). The purpose of comprehensive DGA treatment is to complete dental rehabilitation in a single visit and prevent potential trauma associated with multiple dental visits at a young age (7, 8). However, this goal has not yet been fully achieved. Reportedly, patients have lower relapse rates (24%–54%) in the first 1–6 months following DGA, and the number increases in subsequent visits (53%–80%) (9–14). Moreover, 9%–24% of children are treated with DGA at least twice in their lives (8, 15–17). Several factors may influence the outcomes of DGA treatment. Sheller et al. (18) analyzed factors (treatment- and patient/parent-related factors) that are associated with caries recurrence and repeated DGA in pediatric dentistry patients. They observed that stainless steel crown (SSC) was more reliable than amalgam composite restoration. Besides, the efficiency of the same treatment technique varied in patients with different afflicted tooth types or various risk factors. Patient-related factors included socioeconomic factors, parental compliance with dietary and oral hygiene modifications, time elapsed before follow-up and recall appointments, and exposure to topical or systemic fluoride (18, 19). The occurrence of unplanned retreatment after DGA reflects the outcome of DGA treatment to a certain extent; however, related studies are limited. König et al. (20) observed that the use of fluoride toothpaste significantly reduced the risk of repeated DGA. However, these results were obtained from a parental questionnaire and could not be objectively verified. Therefore, it is critical to summarize the characteristics of unplanned retreatment, examine the possible factors affecting the efficacy of DGA, and find strategies for extending survival time after oral treatment under DGA.

The DGA was performed at the Department of Pediatric Dentistry of the Affiliated Stomatology Hospital of Xi'an Jiaotong University (Shaanxi, China) for approximately 10 years. This hospital was the second institution to provide such treatment in Northwest China. The number of patients undergoing DGA has increased yearly (21), and hospitals’ medical history systems have accumulated a large number of patient records. However, a comprehensive evaluation of the outcomes and unplanned retreatment of patients with DGA has not yet been performed.

The objectives of this study were to (1) retrospectively describe the details of unplanned retreatment of DGA administered to children with S-ECC, (2) assess the survival time after DGA treatment among children with S-ECC, and (3) examine the potential factors that may influence the outcome of DGA.

## 2. Materials and methods

This study was approved by the Medical Ethics Committee of Xi'an Jiaotong University [Xjkqll (2021) no. 10].

### 2.1. Patients

Children who underwent DGA comprehensive treatment in the Department of Pediatric Dentistry, Stomatology Hospital, Xi'an Jiaotong University, between January 1, 2015, and December 31, 2020, were included in the study.

#### 2.1.1. Inclusion criteria

The inclusion criteria include the following: (1) diagnosed with S-ECC; (2) American Society of Anesthesiologists (ASA) grade I (normally healthy without systemic disease) (22); and (3) a post-operative follow-up period of at least 6 months with complete medical records.

#### 2.1.2 Exclusion criteria

The exclusion criteria include the following: (1) DGA treatment for dental trauma and extraction of supernumerary teeth and (2) a follow-up period of less than 6 months.

The deadline for data collection was August 31, 2021. Split-session treatments for multiple problems identified during a single visit were recorded as a single visit.

### 2.2. DGA treatment procedure

All procedures were followed and adjusted according to the related guidelines and textbooks. The patients underwent the following treatments during DGA: (1) prophylactic treatments (e.g., pit and fissure sealants, topical fluoride application); (2) restorative therapy (e.g., filling, preventive resin restoration, and crown restoration such as metal crown and strip resin crown but excluding crown restoration after endodontic treatment); (3) endodontic therapy (e.g., indirect pulp capping, direct pulp capping, pulpotomy, pulpectomy); (4) tooth extractions; and (5) space management.

After DGA treatment, all patients were asked to return for a follow-up visit approximately 14 days later and subsequently every 3–6 months. The patients were required to undergo a radiograph at the first follow-up visit, and other radiographs were taken every 6 months for teeth that underwent endodontic therapy. The outcomes of oral treatment with DGA were reviewed, and timely treatment was administered when necessary. Oral health education was provided to the guardians and children at every follow-up visit. Topical fluoride was applied regularly every 3–6 months, depending on the child's dental caries risk level. Additionally, parents were advised to prepare for over-the-counter non-steroidal anti-inflammatory drugs (NSAIDs) after DGA treatment. Notably, it can take 1–3 days for fever to develop after DGA treatment.

### 2.3. Data collection

Information was obtained from the electronic medical record system. The three parts consisted of the necessary information that was extracted independently by two pediatric dentistry postgraduates using a predesigned form.

The postgraduates were trained to understand the content of the study and the information that needed to be extracted. The consistency test was performed. Moreover, 20 periapical radiographs taken by paralleling technique were submitted to two examiners. They judged the extent to which the root filling material filled the canals in teeth after root canal therapy and repeated it 1 month later. Concordance was assessed by calculating the kappa value in SPSS software (version 22.0; IBM SPSS Statistics, USA). The overall inter- and intra-agreement (kappa) were 0.96 and 0.91, respectively.

(1) Background information: case number, sex, date of birth, and date of treatment.
(2) Treatment information: treated tooth position, diagnosis, treatments received, and the total number of treated teeth. For one tooth, multiple treatments were allowed but were only counted once (e.g., teeth that underwent metal crown restoration after pulpectomy were only recorded as pulpectomies).
(3) Follow-up information: follow-up date, number of follow-up visits, whether the patient received unplanned retreatment, and other details. The frequency of fluoride application during the follow-up period was also recorded.

The reasons for unplanned retreatment included (1) restoration failure: crown/filling fracture/cracking or falling out without dental caries; (2) secondary caries; (3) new caries, the occurrence of caries on the healthy tooth surface after pit and fissure sealing or filling; (4) dental pulp diseases; (5) periapical periodontitis; (6) tooth fracture without caries; and (7) early loss of the primary teeth.

### 2.4. Data analysis

All data were tabulated in Microsoft Excel 2019, and a digital database was created. Survival time was calculated based on the treatment date under DGA and the date of the first unplanned retreatment. The number and frequency (times/year) of follow-up visits and fluoride applications were recorded and calculated. The longest follow-up period was calculated from the date of DGA treatment until the last visit date. The total number of visits, fluoride applications, and frequency (times/year) were recorded and calculated.

The SPSS software (version 22.0; IBM SPSS Statistics, USA) was used to categorize and analyze the data. Normally distributed data are described as mean ± standard deviation (SD). Data with skewed distribution are described as medians, and count data are expressed as frequencies and proportions. The Mann–Whitney *U*-test was used to compare the medians between the two groups, and the *χ*^2^ test was used to test the proportion distribution differences. The R software (version 4.04) was used to calculate survival rates and draw curves at different times after treatment using the Kaplan‒Meier (KM) method. The Cox proportional hazards model was used for further analysis to explore the predictors of unplanned retreatment among factors with significant associations. Statistical significance was set at *P* < 0.05.

## 3. Results

**Figure 1:**
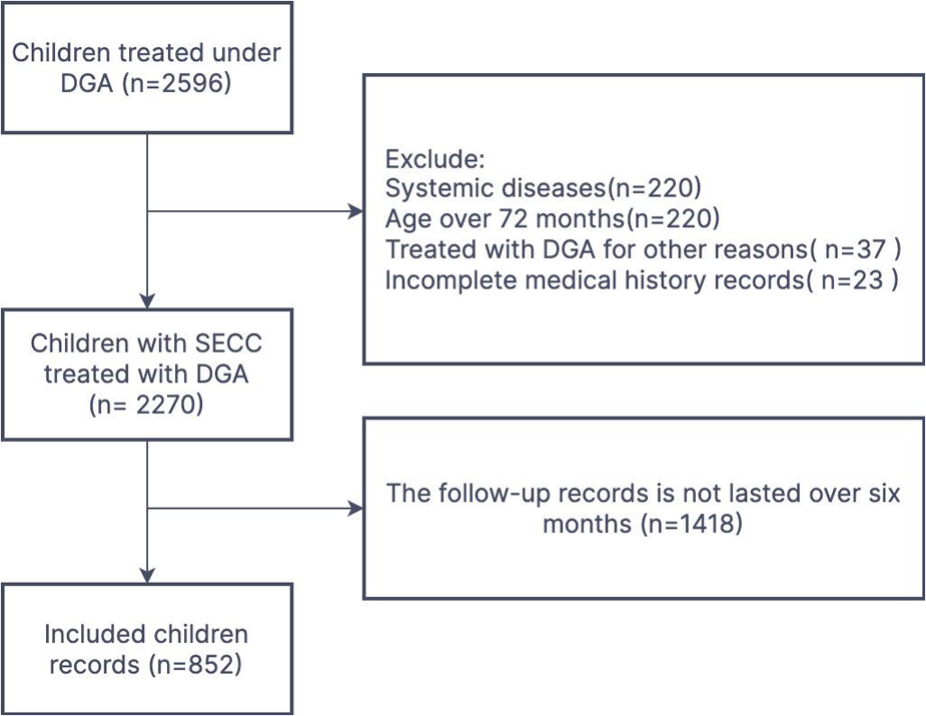
Flowchart of the screening of eligible children.

From January 1, 2015, to December 31, 2020, a total of 2,270 children with S-ECC without systemic disease underwent DGA treatment. Only 852 (37.5%) patients had a complete follow-up visit record lasting over 6 months and were included in this study (Figure 1). The gender of the children was roughly an equal split (437 boys, 416 girls), with a mean age of 3.4 ± 0.9 years. A total of 12,111 teeth were subjected to different treatments. Indirect pulp capping (25%) was the most common treatment for DGA, followed by restoration (23%) and pulpectomy (21%). An average of 14 ± 3 teeth per child was treated. The longest follow-up time was 2,118 days (5.8 years), median follow-up time was 511 days (1.4 years), and frequency of follow-up visits ranged from 0.4 to 4.6 times/year, with an average of 1.8 ± 0.8 times/year.

### 3.1. Unplanned retreatment during the whole period

A total of 509 (59.7%) children and 1,212 (10.7%, 1,212/11,377, excluding the extracted teeth) teeth were subjected to unplanned retreatment. Only one child received repeated DGA treatment, 18 received retreatment via protective stabilization, and the remaining children were retreated in a normal setting. An average of 2.39 (1,212/509) teeth per child were retreated.

The incidence of treatment failure of the anterior teeth (12.8%, 643/5,043) was significantly higher than that of the posterior teeth (9.0%, 569/6,322) (*P *< 0.001). The treatment failure rate of the upper teeth (12.6%, 854/6,778) was significantly higher than that of the lower teeth (7.8%, 358/4,590) (*P *< 0.001). The left teeth failed more frequently than the right teeth (11.3% vs. 10.0%, 643/5,690 vs. 569/5,690, *P *< 0.05). The upper central incisor was the most likely tooth to experience treatment failure (18.3%, 217/1,187).

The reasons for retreatment at the tooth level included restoration failure (30.12%, 365/1,212), new caries (29.46%, 357/1,212), and periapical periodontitis (22.44%, 272/1,212). Tooth fractures occurred in only two cases.

The incidences of restoration failure and secondary caries in anterior teeth were higher than those in posterior teeth (all *P *< 0.05). The posterior teeth were more prone to new caries; however, the interval difference was not significant. The occurrence of pulp disease showed a higher rate in posterior teeth, but the difference in periapical periodontitis between anterior and posterior teeth was not significant (Table 1). The incidence and details of the different reasons for failure are presented in the Appendix.

**Table 1:** Distribution and failure time of different reasons for retreatment between anterior and posterior teeth.

	Overall incidence	Tooth position	Tooth number	Incidence	*P* ^a^	Median failure time (days)	*P* ^b^
Restoration failure	3.38%	Anterior	313	6.21%	<0.05	274	<0.05
		Posterior	52	0.81%		355.5	
New caries	4.61%	Anterior	85	2.33%	<0.05	554	0.45
		Posterior	272	5.50%		612	
Secondary caries	1.24%	Anterior	112	2.22%	<0.05	362	<0.05
		Posterior	29	0.46%		584	
Periapical periodontitis	2.39%	Anterior	118	2.34%	0.75	647	0.41
		Posterior	154	2.43%		673	
Pulp disease	0.6%	Anterior	11	0.28%	<0.05	631	0.22
		Posterior	42	0.84%		703	

The sample size for retreatment analysis: 11,377 (5,043 anterior teeth, 6,334 posterior teeth).

^a^ Chi-square test.

^b^ Mann-Whitney *U*-test.

### 3.2. Survival analysis of DGA treatment

During the entire follow-up period, the KM analysis results showed that the median survival time of the 852 children was 510 days (1.4 years). From the perspective of the child level, survival rates at 6, 12, 18, 24, 30, and 36 months were 87.6%, 64.6%, 47.1%, 31.9%, 19.7%, and 10.7%, respectively. At the tooth level, the median survival time was 1,911 days (5.2 years). Survival rates at 6, 12, 24, 36, 48, and 60 months were 98.7%, 91.8%, 86.9%, 74.3%, 63.5%, and 54.4%, respectively.

### 3.3. Exploring potential factors

Sex, age at the time of DGA, frequency of revisits, and fluoride application at the first unplanned retreatment were considered potential factors that may influence the success rate of DGA treatment. Except for sex, all other variables showed a significant influence on the DGA success rate in the univariate analysis (*P *< 0.05) (Table 2).

**Table 2:** Univariate analysis of potential factors affecting the DGA treatment success rate.

Factors	*P*	HR	95% CI	
			Lower	Upper
Gender: female	0.440	0.934	0.784	1.111
Age (months)	<0.001	0.973	0.965	0.981
Revisit frequency	<0.001	0.773	0.681	0.878
Topical fluoride application frequency	<0.001	0.706	0.587	0.848

HR, hazard ratio; CI, confidence interval.

Revisit frequency (times per year) = total revisit times/total revisit period.

Topical fluoride application frequency (times per year) = total application times/total revisit period.

As the revisit frequency and fluoride application frequency were not mutual, they were placed separately with age at the time of DGA in the multivariate analysis. The results are summarized in Table 3. The results showed that older S-ECC children treated with DGA, or those with a higher frequency of follow-up and fluoride application, had a lower risk of post-operative unplanned retreatment (HR = 0.97, 0.78, 0.69, *P *< 0.001).

**Table 3:** Multivariate analysis of potential factors affecting the DGA treatment success rate.

Factors	*P*	HR	95%CI	
			Lower	Upper
Age (months)	<0.001	0.974	0.965	0.982
Revisit frequency	<0.001	0.791	0.697	0.897
Age (months)	<0.001	0.972	0.964	0.980
Topical fluoride application frequency	<0.001	0.686	0.571	0.824

HR, hazard ratio; CI, confidence interval.

Revisit frequency (times per year) = total revisit times/total revisit period.

Topical fluoride application frequency (times per year) = total application times/total revisit period.

## 4. Discussion

DGA technology has gradually been accepted by more and more pediatric dentists over the years. Children who cannot cooperate with dentists or those who require extensive treatment can access effective restorative care under DGA. However, the treatment regimen under DGA varies by country, medical institution, dentist, and subject. In some Western countries, tooth filling and extraction are the main options during DGA (8, 16); nevertheless, studies conducted in China showed that endodontic treatments accounted for a large proportion of the total treatments and were performed more than extractions (23, 24). In this study, direct or indirect pulp treatments comprised approximately half (48.9%), while tooth extractions accounted for only 6.06% of the treated teeth. Chinese pediatric dentists were more likely to choose tooth conservation for primary teeth, and the treatment regime was more conservative.

The responsibility of pediatric dentists includes not only rehabilitating the patient’s oral health but also educating them to establish healthy oral habits, achieving a long-term dental caries-free status. Studies have shown that DGA treatment can significantly reduce the activity of dental caries in a short period; however, this effect cannot be maintained permanently (25). For instance, one study observed a significant reduction in cariogenic microorganisms after DGA treatment, but it also showed a tendency to increase over time (26). Batawi (12) and König et al. (20) concluded that dental caries relapse is correlated with patients’ compliance. In this retrospective study, only 37.5% of patients revisited at least 6 months after DGA treatment, indicating that guardians’ awareness of post-operative preventive healthcare was insufficient. A systematic review showed that oral health education can significantly improve oral hygiene, knowledge levels, and attitudes toward oral behaviors (27). Therefore, dentists should emphasize the importance of regular patient revisits and provide instructions for maintaining oral hygiene. Guardians should also be informed that DGA treatment can fail quickly if dental neglect is prolonged.

In this study, the overall retreatment rate was 59.7% at the child level and 10.79% at the tooth level, which is similar to the results of other studies (11–14). However, the incidence of repeat DGA is much lower than that in other countries, which may be attributed to the fact that DGA treatment is relatively expensive and is not covered by insurance (8, 15–17). A reasonable treatment regimen and thorough preoperative examination also result in a low repeat DGA rate (16). For example, one study confirmed that full coverage of all second primary molars with SSC in DGA is associated with a lower risk of repeated DGA (28). Therefore, a comprehensive preoperative examination should be implemented before a DGA recommendation, including general status, oral status, oral health-related behavior, and sociodemographic factors. A thorough examination can help the dentist to select the optimal treatment plan. For instance, the sociodemographic background gives indications of revisiting possibility for pediatric patients (18). A more aggressive treatment plan may be more suitable considering the overall cost, potential complications, and retreatment risks.

Among the retreatment teeth, restoration failure accounted for the biggest proportion. The retreatment rate for restoration in anterior teeth was significantly higher than in posterior teeth. A possible explanation is that the anterior teeth are involved in caries more often than the posterior teeth because of an earlier eruption time, especially in children under 3 years old. Thus, by the time children received DGA treatment, anterior tooth decay could already be in advanced extension, severely damaging the tooth structure. This results in poor retention of the restorative material. A previous study showed that the success rate of resin restoration was significantly lower than that in posterior teeth (29). These findings indicated that there were different critical aspects for the restoration of anterior teeth and posterior teeth. As for anterior teeth, pediatric dentists should particularly pay attention to the indications and retain healthy dental tissue as much as possible. The most important is that dentists are supposed to improve their restorative procedures, for instance, tooth preparation, standardized adhesion steps, and saliva isolation. Regarding posterior teeth, SSC application under DGA has a higher survival rate than that of composite restorations (30, 31). Routinely using metal crowns is strongly recommended to restore primary molars treated with endodontics or complex filling, especially for children who may have poor attendance to post-operative follow-ups (18, 20). For routine pediatric outpatient, the Hall technique can be an alteration for SSC because of its high success rate and shorter treatment time (32, 33).

From the perspective of new caries, the incidence in posterior teeth was also higher than that in anterior teeth, mainly caused by the 102 posterior teeth treated with pit and fissure sealing. Although a study has shown that the incidence of dental caries in teeth treated with pit and fissure sealing can be reduced by 11%–51%, the rate of average sealant loss can reach 40% (34, 35). Partial detachment of the sealant can induce plaque accumulation, contribute to difficulty in cleaning, and increase the risk of tooth decay. In this study, 17.96% of the teeth that underwent pit and fissure occlusion had new caries. It is suggested that during DGA, dentists should try to cover only pits and fissures and avoid excessive sealant to prevent partial loss of the sealant. Rubber dam and occlusion adjustment should be routinely used under DGA. Preventive recall appointment is encouraged for re-examination, knowledge reinforcement, and addressing some early diseases.

In the survival analysis, the median survival time was 510 days at the child level, indicating that half of the children required unplanned retreatment 1 year and 5 months after DGA treatment. The survival rate gradually decreased, and the 36-month survival rate was 10.7%. However, the average number of retreated teeth was only 1.4 (1,212/825) per patient. The 5-year survival rate remained >50% at the tooth level. Considering that the functional time of primary incisors is 5–8 years and that of canines and molars is 8–10 years, treatment under DGA can satisfy the functional duration for most teeth without clinical symptoms (36). Moreover, other studies related to the effects of DGA have also proved that the life quality of S-ECC children can be improved after DGA (37, 38). Therefore, the clinical outcomes and psychological effects of DGA in children were satisfactory.

The present multivariate analysis revealed that the risk of retreatment decreased with increasing age at the time of DGA, which is consistent with the findings of Amin et al. (11). When children were treated with DGA, the posterior teeth had not completely erupted or they had just mild caries. They required only pit and fissure sealing or preventive resin filling instead of crown restoration; however, these simple treatments may cause potential problems. Older children have a higher degree of cooperation in post-operative oral healthcare, which is beneficial in maintaining treatment efficacy. The current study observed that reasonably enhancing the frequency of follow-up and topical fluoride application could lower the risk of retreatment, which is consistent with the results of Batawi (12) and Amin et al. (11, 19). These results illustrated the significance of regular follow-up visits and medication treatment.

Even though in the present study, we focused on the dental treatment of S-ECC under DGA, we need to clearly recognize that DGA should not be the first option for children. DGA is considered a traumatic treatment for children because of the fear during anesthesia induction (39). Some clinicians and researchers have reported that children who had experienced DGA have greater dental anxiety than those without a history of DGA (40, 41). A recent study by Zhou et al. showed that DGA caused a higher risk for dental anxiety when compared with that under physical restraints in the long term, which may lead to uncooperative dental behavior (39). Besides, post-operative complications following treatment under DGA should also be paid real attention to. According to Zhang et al., approximately 94.86% of the enrolled children who received DGA reported one or more complications. The most prevalent complication was post-operative pain (42). The results in our current study also show that 509 (59.7%) children with 1,212 (10.7%) teeth underwent unplanned retreatment after DGA. These would undoubtedly aggravate children's discomfort and parents’ anxiety.

Except for non-pharmacological guidance techniques, today there are alternatives to control caries disease and allow the child to mature to accept conventional treatment where it is needed. The announcement of breakthrough therapy designation by the Food and Drug Administration (FDA) suggests that silver diamine fluoride (SDF) may become the first FDA-approved drug for treating caries (43). Based on the best available evidence, a logical approach can be adopted regarding the practical use of 38% SDF for caries prevention and arrest (44, 45). When SDF is applied to active lesions, it can be used with or without subsequent restoration, depending on clinical context, expert judgment, and patient input. However, the non-invasive treatments by medication are invalid for teeth with a large cavitated lesion or pulp inflammation (45), and could not rehabilitate tooth integrity. Therefore, medication is a good option to prevent caries from rapidly progressing for S-ECC patients while children are waiting for more treatment.

It is undeniable that this study has few limitations. First, the sociodemographic and socioeconomic factors and oral health-related behavior were not included in the analysis. Dental caries is a multifactorial disease, and occurrence and relapse risks should be assessed from different perspectives. However, because of the retrospective design of the study, it was difficult to collect background information on the families of the enrolled children. In addition, in the present medical history record system, no information about the background of the families or oral health behaviors was recorded. Second, all medical records came from one public hospital, and the inclusion criteria limited the enrolled children to those without systemic diseases. Therefore, the representativeness of the results of this study may have led to bias and reduced generalizability. Lastly, a proportion of people who lived far away had limited regular access to the hospital during follow-up and may have received unplanned retreatment during the follow-up period anywhere else. Thus, the retreatment rate may be underestimated, and the success rate may not be accurate.

## 5. Conclusions

The treatment outcome of DGA administered to children with S-ECC was satisfactory at the tooth level; however, the proportion of children receiving unplanned retreatment was high, and restoration failure was the main reason. Strategies for improving the outcome of DGA include medical team and parental/patient aspects. Pediatric dentists should improve their medical knowledge and skills to provide optimal treatment service and thorough dental health education for patients. Enhancing parental compliance and increasing the frequency of topical fluoride application within a reasonable range may improve the success rate of DGA treatment.

## Data Availability

The raw data supporting the conclusions of this article will be made available by the authors, without undue reservation.

## References

[1] Kazeminia M Abdi A Shohaimi S Jalali R Vaisi-Raygani A Salari N Dental caries in primary and permanent teeth in children's worldwide, 1995 to 2019: a systematic review and meta-analysis. Head Face Med. (2020) 16(1):22. 10.1186/s13005-020-00237-z 33023617 PMC7541284

[2] Finucane D. Rationale for restoration of carious primary teeth: a review. Eur Arch Paediatr Dent. (2012) 13(6):281–92. 10.1007/bf03320828 23235127

[3] Sheiham A. Dental caries affects body weight, growth and quality of life in pre-school children. Br Dent J. (2006) 201(10):625–6. 10.1038/sj.bdj.4814259 17128231

[4] Wen PYF Chen MX Zhong YJ Dong QQ Wong HM. Global burden and inequality of dental caries, 1990 to 2019. J Dent Res. (2022) 101(4):392–9. 10.1177/00220345211056247 34852668

[5] American Academy on Pediatric Dentistry; American Academy of Pediatrics. Policy on early childhood caries (ECC): classifications, consequences, and preventive strategies. Pediatr Dent. (2016) 38(6):52–4. 27931420

[6] American Academy on Pediatric Dentistry Clinical Affairs Committee-Behavior Management Subcommittee; American Academy on Pediatric Dentistry Council on Clinical Affairs. Guideline on behavior guidance for the pediatric dental patient. Pediatr Dent. (2016) 38(6):185–98. 27931459

[7] Mittal R Sharma M. Assessment of psychological effects of dental treatment on children. Contemp Clin Dent. (2012) 3(Suppl 1):S2–7. 10.4103/0976-237x.95093 22629059 PMC3354802

[8] Savanheimo N Vehkalahti MM. Five-year follow-up of children receiving comprehensive dental care under general anesthesia. BMC Oral Health. (2014) 14:154. 10.1186/1472-6831-14-154 25512015 PMC4277839

[9] Berkowitz RJ Moss M Billings RJ Weinstein P. Clinical outcomes for nursing caries treated using general anesthesia. ASDC J Dent Child. (1997) 64(3):210–1, 228. 9262804

[10] Chase I Berkowitz RJ Mundorff-Shrestha SA Proskin HM Weinstein P Billings R. Clinical outcomes for early childhood caries (ECC): the influence of salivary mutans streptococci levels. Eur J Paediatr Dent. (2004) 5(3):143–6. 15471521

[11] Amin M Nouri R ElSalhy M Shah P Azarpazhooh A. Caries recurrence after treatment under general anaesthesia for early childhood caries: a retrospective cohort study. Eur Arch Paediatr Dent. (2015) 16(4):325–31. 10.1007/s40368-014-0166-4 25619862

[12] Batawi HYE. Factors affecting clinical outcome following treatment of early childhood caries under general anaesthesia: a two-year follow-up. Eur Arch Paediatr Dent. (2014) 15(3):183–9. 10.1007/s40368-013-0081-0 24030856

[13] Drummond BK Davidson LE Williams SM Moffat SM Ayers KM. Outcomes two, three and four years after comprehensive care under general anaesthesia. N Z Dent J. (2004) 100(2):32–7. 15346870

[14] Lin YT Lin YJ. Factors associated with the risk of caries development after comprehensive dental rehabilitation under general anesthesia. J Dent Sci. (2016) 11(2):164–9. 10.1016/j.jds.2016.01.004 30894966 PMC6395190

[15] Almeida AG Roseman MM Sheff M Huntington N Hughes CV. Future caries susceptibility in children with early childhood caries following treatment under general anesthesia. Pediatr Dent. (2000) 22(4):302–6. 10969437

[16] Karl V Scholz KJ Hiller KA Tabenski I Schenke F Buchalla W Retrospective cohort study on potential risk factors for repeated need of dental rehabilitation under general anesthesia in a private pediatric dental practice. Children (Basel). (2022) 9(6):855. 10.3390/children9060855 35740792 PMC9221647

[17] Schroth RJ Smith WF. A review of repeat general anesthesia for pediatric dental surgery in Alberta, Canada. Pediatr Dent. (2007) 29(6):480–7. 18254418

[18] Sheller B Williams BJ Hays K Mancl L. Reasons for repeat dental treatment under general anesthesia for the healthy child. Pediatr Dent. (2003) 25(6):546–52. 14733468

[19] Amin MS Bedard D Gamble J. Early childhood caries: recurrence after comprehensive dental treatment under general anaesthesia. Eur Arch Paediatr Dent. (2010) 11(6):269–73. 10.1007/BF03262761 21108916

[20] König T Reicherts P Leha A Hrasky V Wiegand A. Retrospective study on risk factors for repeated dental treatment of children under general anaesthesia. Eur J Paediatr Dent. (2020) 21(3):183–6. 10.23804/ejpd.2020.21.03.04 32893648

[21] Liu F Yang K Wang P Wu T Li J Trends GQ. Characteristics, and success rates of treatment for severe early childhood caries under general anesthesia: a retrospective study in Northwest China. J Clin Pediatr Dent. (2021) 45(4):278–83. 10.17796/1053-4625-45.4.11 34534298

[22] Doyle DJ Goyal A Garmon EH. American Society of Anesthesiologists classification. Statpearls. Treasure Island, FL: StatPearls Publishing (2022). 28722969

[23] Chen YP Hsieh CY Hsu WT Wu FY Shih WY. A 10-year trend of dental treatments under general anesthesia of children in Taipei veterans general hospital. J Chin Med Assoc. (2017) 80(4):262–8. 10.1016/j.jcma.2016.11.001 28100415

[24] Xia B Qin M Ma WL Liu H Wang JH Liu KY A retrospective study of 693 children's dental treatment under general anesthesia. Beijing Da Xue Xue Bao Yi Xue Ban. (2013) 45(6):984–8. 24343087

[25] Zhao J Yang L Lai G Wang J. Clinical outcomes of dental treatment under general anesthesia and its effects on the caries activity and body growth of children: a 2-year retrospective study. Clin Oral Investig. (2022) 26(5):4091–8. 10.1007/s00784-022-04377-1 35118521

[26] Klinke T Urban M Lück C Hannig C Kuhn M Krämer N. Changes in Candida spp., mutans streptococci and lactobacilli following treatment of early childhood caries: a 1-year follow-up. Caries Res. (2014) 48(1):24–31. 10.1159/000351673 24216710

[27] Habbu SG Krishnappa P. Effectiveness of oral health education in children—a systematic review of current evidence (2005–2011). Int Dent J. (2015) 65(2):57–64. 10.1111/idj.12137 25345565 PMC9376487

[28] Azadani EN Casamassimo PS Peng J Griffen A Amini H Kumar A. Primary second molar treatment as a predictor of repeat general anesthesia. Pediatr Dent. (2021) 43(5):380–6. 34654500

[29] Lin Y-T Lin Y-TJ. Survey of comprehensive restorative treatment for children under general anesthesia. J Dent Sci. (2015) 10(3):296–9. 10.1016/j.jds.2014.09.002

[30] Mallineni SK Yiu CK. A retrospective review of outcomes of dental treatment performed for special needs patients under general anaesthesia: 2-year follow-up. ScientificWorldJournal. (2014) 2014:748353. 10.1155/2014/748353 25610913 PMC4290790

[31] Al-Eheideb AA Herman NG. Outcomes of dental procedures performed on children under general anesthesia. J Clin Pediatr Dent. (2003) 27(2):181–3. 10.17796/jcpd.27.2.k3307186n7086r11 12597693

[32] Ebrahimi M Shirazi AS Afshari E. Success and behavior during atraumatic restorative treatment, the Hall technique, and the stainless steel crown technique for primary molar teeth. Pediatr Dent. (2020) 42(3):187–92. 32522320

[33] Ludwig KH Fontana M Vinson LA Platt JA Dean JA. The success of stainless steel crowns placed with the Hall technique: a retrospective study. J Am Dent Assoc. (2014) 145(12):1248–53. 10.14219/jada.2014.89 25429038

[34] Ahovuo-Saloranta A Forss H Walsh T Nordblad A Mäkelä M Worthington HV. Pit and fissure sealants for preventing dental decay in permanent teeth. Cochrane Database Syst Rev. (2017) 7(7):CD001830. 10.1002/14651858.CD001830.pub5 28759120 PMC6483295

[35] Beun S Bailly C Devaux J Leloup G. Rheological properties of flowable resin composites and pit and fissure sealants. Dent Mater. (2008) 24(4):548–55. 10.1016/j.dental.2007.05.019 17659769

[36] Kratunova E Silva D. Pulp therapy for primary and immature permanent teeth: an overview. Gen Dent. (2018) 66(6):30–8. 30444704

[37] Duruk G Kuru R Özkan AS. Impact of dental rehabilitation under general anesthesia on oral health-related quality-of-life and dental anxiety in Turkish children. Pesquisa Bras Odontopediatria e Clín Integr. (2020) 21:e0109. 10.1590/pboci.2021.006

[38] Anderson HK Drummond BK Thomson WM. Changes in aspects of children's oral-health-related quality of life following dental treatment under general anaesthesia. Int J Paediatr Dent. (2004) 14(5):317–25. 10.1111/j.1365-263X.2004.00572.x 15330997

[39] Zhou F Zhang S Ma W Xiao Y Wang D Zeng S The long-term effect of dental treatment under general anaesthesia or physical restraints on children’s dental anxiety and behaviour. Eur J Paediatr Dent. (2022) 23(1):27–32. 10.23804/ejpd.2022.23.01.05 35274539

[40] Aldossari GS Aldosari AA Alasmari AA Aldakheel RM Al-Natsha RR Aldossary MS. The long-term effect of previous dental treatment under general anaesthesia on children’s dental fear and anxiety. Int J Paediatr Dent. (2018). 10.1111/ipd.12455. [Epub ahead of print] 30506997

[41] Haworth S Dudding T Waylen A Thomas SJ Timpson NJ. Ten years on: is dental general anaesthesia in childhood a risk factor for caries and anxiety? Br Dent J. (2017) 222(4):299–304. 10.1038/sj.bdj.2017.175 28232699 PMC5565940

[42] Zhang Q Deng X Wang Y Huang R Yang R Zou J. Postoperative complications in Chinese children following dental general anesthesia: a cross-sectional study. Medicine (Baltimore). (2020) 99(45):e23065. 10.1097/md.0000000000023065 33157964 PMC7647524

[43] Horst JA. Silver fluoride as a treatment for dental caries. Adv Dent Res. (2018) 29(1):135–40. 10.1177/0022034517743750 29355428 PMC6699125

[44] Young DA Quock RL Horst J Kaur R MacLean JK Frachella JC Clinical instructions for using silver diamine fluoride (SDF) in dental caries management. Compend Contin Educ Dent. (2021) 42(6):e5–9. 34412482

[45] American Academy on Pediatric Dentistry; American Academy of Pediatrics. Policy on the use of silver diamine fluoride for pediatric dental patients. Pediatr Dent. (2018) 40(6):51–4. 32074849

