# Supplementary material for: Incidence and risk factors of unplanned retreatment following dental general anesthesia in children with severe early childhood caries
Source: Front Pediatr. 2023 Jul 27;11:1163368. doi: 10.3389/fped.2023.1163368 (PMC10413873; doi:10.3389/fped.2023.1163368)
Supplement: Supplementary file 1 [file Table1.docx]

Supplementary Material

Incidence of Unplanned Retreatment of Dental General Anesthesia Treatment and Risk Factors Given to Children with Severe Early Childhood Caries

Jin-yi Li ^1,2#^, Shu-yang He ^3#^, Pan-xi Wang ^1,2^, Shan-shan Dai ^1,2^, Shu-qi Zhang ^1,2^, Zheng-yang Li ^1,2^, Qing-yu Guo ^1,2*^, Fei Liu ^1,2*^

# These authors are contributed equally to this work and shared the first author.

*** Correspondence:**Qing-yu Guo and Fei Liu
guoqinyu@mail.xjtu.edu.cn (QG); liufei6630@mail.xjtu.edu.cn (FL)

**Appendix Table 1 The details and incidence of different failure outcomes**

**underwent different treatments**

|  | Filling (n, %) | Crown restoration (n, %) | Vital pulp therapy (n, %) | Pulpectomy (n, %) | Pit and fissure sealing (n, %) | *P* |
| --- | --- | --- | --- | --- | --- | --- |
| Restoration failure | 16 (0.76) | 53 (1.90) | 72 (2.1) | 220 (8.83) | ___ | ***< 0.05*** |
| New caries | 173 (8.26) | ___ | 72 (2.1) | 10 (0.40) | 102 (17.96) | ***< 0.05*** |
| Secondary caries | 25 (1.19) | 15 (0.54) | 31 (0.90) | 70 (2.81) | ___ | ***< 0.05*** |
| Periapical periodontitis | 15 (0.72) | 38 (1.36) | 63 (1.84) | 153 (6.14) | 3 (0.53) | 0.357 |
| Pulp disease | 22 (1.05) | 7 (0.25) | 14 (0.41) | ___ | 9 (1.58) | ***< 0.05*** |
| Tooth early loss | ___ | ___ | ___ | 12 (0.48) | ___ | ___ |
| Tooth facture | ___ | ___ | ___ | 2 (0.00) | ___ | ___ |

Percentage in the brackets are the incidence

P value calculated by Chi-square test.

**
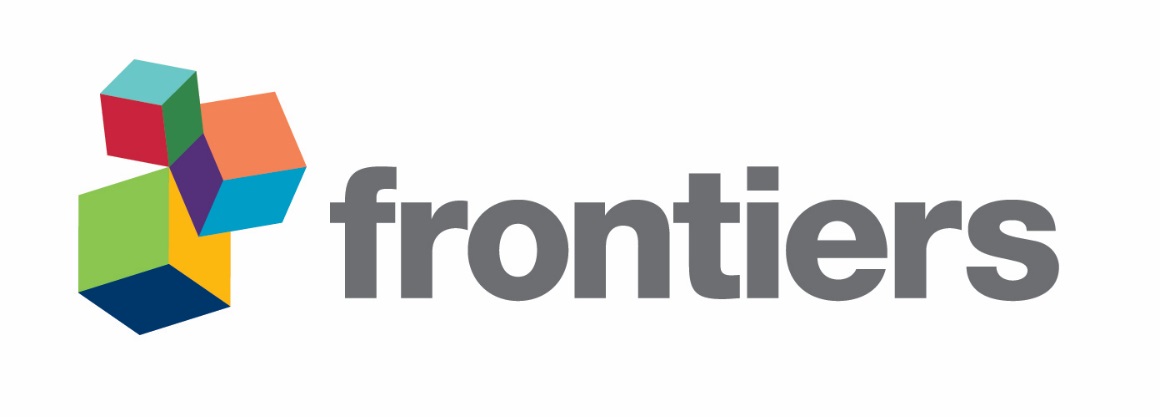
**
